# Platelet count polygenic scores may modify the effect of aspirin in primary prevention of dementia

**DOI:** 10.1002/alz.71817

**Published:** 2026-09-24

**Authors:** Peter D. Fransquet, Chenglong Yu, Cammie Tran, Sultana Monira Hussain, Amy Brodtmann, Andrew M. Tonkin, Mark R Nelson, Joanne Ryan, John J. McNeil, Paul Lacaze

**Affiliations:** ^1^ School of Public Health and Preventive MedicineFaculty of Medicine, Nursing and Health Sciences Monash University Melbourne Victoria Australia; ^2^ Victorian Heart Institute Monash University Clayton Melbourne Australia; ^3^ Department of Neuroscience School of Translational Medicine, Faculty of Medicine, Nursing and Health Sciences Monash University Melbourne Victoria Australia; ^4^ Menzies Institute for Medical Research University of Tasmania Hobart Australia

**Keywords:** aspirin, ASPREE trial, dementia prevention, genetic risk stratification, pharmacogenomics, platelet count, polygenic risk score, precision medicine

## Abstract

**INTRODUCTION:**

Aspirin is not recommended for dementia prevention due to limited benefit. We investigated whether genetic subgroups may benefit.

**METHODS:**

In the ASPirin in Reducing Events in the Elderly (ASPREE) trial (*n* = 13,541), we screened 5182 polygenic risk scores (PGSs) for aspirin interactions on dementia and major bleeding. After quality control, 1848 PGSs were analyzed using Cox models for aspirin×PGS interaction, adjusted for age, sex, *apolipoprotein E*status, and ancestry. Interactions were Bonferroni‐corrected and examined by quintiles.

**RESULTS:**

Platelet‐related PGS accounted for 18/112 nominally significant interactions (enrichment_OR = 54.7, *p* = 3.1 × 10^−^^1^ ^8^). Three highly correlated platelet‐count PGSs passed multiple testing. For participants in the highest quintile of the platelet‐count PGS003548, aspirin allocation was associated with a 3.5‐fold reduction in incident dementia versus placebo (hazard ratio [HR]: 0.28, 95% confidence interval [CI]: 0.15 to 0.52). Major bleeding was also increased (HR: 2.13, 95% CI: 1.36 to 3.34). Similar interactions were observed using the highest PGS003548 tertile, decile, and 5%.

**DISCUSSION:**

Platelet count‐related genetic variation may modify aspirin effects on dementia. These hypothesis‐generating findings require validation.

## Highlights

- Platelet‐count PGSs modify aspirin effects on dementia risk.
- Aspirin reduced dementia risk 3.5‐fold in the highest platelet genetic quintile.
- No dementia benefit was observed in the remaining genetic quintile subgroups.
- However, aspirin increased major bleeding risk in the high genetic subgroup 2.1‐fold.
- Findings support precision prevention using genetic stratification.

## 1 BACKGROUND

There are no medications currently approved for the primary prevention of dementia.^1^ The prevention of dementia therefore continues to focus largely on non‐pharmacological and modifiable risk factors.^2^ Given the aging global population and the increase in dementia prevalence, particularly in those of lower socioeconomic status,^3^ pharmacological strategies that could reduce or delay dementia onset are of considerable public health interest, particularly if cheap and accessible.

The ASPirin in Reducing Events in the Elderly (ASPREE) trial was a large randomized controlled trial (RCT) involving daily low‐dose aspirin in relatively healthy older adults. The trial previously reported no overall effect of aspirin on dementia.^4^ ASPREE was among the largest RCTs of older people ever completed (19,114 participants) and included dementia as a pre‐specified trial endpoint. The lack of an overall aspirin effect on dementia risk in the ASPREE trial^4^ was based on analysis of average effects across the entire cohort and did not account for potential individual heterogeneity in treatment response or genetic subgrouping. However, the inclusion of dementia outcomes and availability of genomic data in ASPREE provide a unique setting for post‐hoc genetic stratification analysis to explore possible effect‐modifications of aspirin treatment.

Inter‐individual variability in dementia risk is well recognized, with genetic variability, particularly with the *apolipoprotein E* *(APOE)* ε4 allele, playing a substantial role in susceptibility.^5^ Beyond single genetic loci, polygenic risk scores (PGSs) comprising thousands of different variants, now also offer a broader, genome‐wide measure of an individual's inherited risk for a wide range of traits and diseases. Such polygenic scores have potential applications in stratifying risk, uncovering heterogeneity in treatment response, and increasingly in identifying subgroups that may benefit from specific interventions.

Aspirin has a range of anti‐platelet, anti‐thrombotic, and possibly anti‐inflammatory effects.^6^, ^7^ Therefore, it is biologically plausible that polygenetic pathways influencing neurodegeneration, cerebrovascular function, platelet biology, or inflammatory regulation may modify the effect of aspirin on dementia risk. We aimed to undertake a hypothesis‐generating study, screening multiple existing PGSs that may help identify subgroups who respond differently to aspirin in the setting of dementia prevention. We undertook a comprehensive screen of all available polygenic scores from the PGS Catalog,^8^ to evaluate whether any PGS may modify the effects of aspirin on dementia in the ASPREE trial. Further, because low‐dose aspirin is also associated with an increased risk of bleeding in older people,^9^ we further examined whether the identified PGS also interacted with major bleeding outcomes (harms) in the trial, to assess benefit versus harm, after polygenetic stratification.

## 2 METHODS

### 2.1 Study design and population

We conducted a post hoc genetic analysis of genotyped participants in the ASPREE trial, a randomized, placebo‐controlled study of 100 mg daily low‐dose aspirin versus placebo in healthy older adults. At baseline, all participants were 70 years of age or older in Australia and the United States (65 years or older for US minorities). Eligible participants had no prior history of cardiovascular disease events, dementia, physical disability, or chronic disease likely to limit survival to less than 5 years, and all had a Modified Mini‐Mental State Examination (3MS) score > 78.^10^ Continuous use of antiplatelet or anticoagulant drugs was also a study exclusion criterion. All participants provided written informed consent. Of the 19,114 participants, bio‐samples (blood/saliva) for genotyping were available for 14,117 individuals who consented to genetic analysis and had suitable DNA.^11^ The ASPREE trial was approved by the Human Research Ethics Committees at Monash University and Alfred Hospital (Australia) and by Institutional Review Boards at US sites and was registered at ClinicalTrials.gov (NCT01038583). This study was performed in accordance with the ethical standards of the 1964 Declaration of Helsinki and its later amendments.

### 2.2 Endpoints

**RESEARCH IN CONTEXT**

1. **Systematic review**: We searched the literature on aspirin and dementia prevention, including randomized trials and observational studies, and reviewed genetic studies of dementia risk and platelet biology. Prior evidence, including ASPREE, shows no overall benefit of aspirin for dementia prevention, and no studies have systematically evaluated polygenic modification of aspirin's effects.
2. **Interpretation**: In this pharmacogenomic analysis, platelet‐related PGSs identified a subgroup in which aspirin was associated with reduced dementia risk, alongside increased bleeding risk. These findings suggest platelet biology may underlie heterogeneity in aspirin response and dementia risk.
3. **Future directions**: Replication in independent cohorts and trials is required. Studies integrating genetic risk with measured platelet function and neurovascular markers and evaluating clinical utility for precision prevention are needed.

The primary endpoints for this study were dementia and major bleeding. These events were adjudicated by expert committees, blinded to treatment allocation, as previously described.^4^, ^9^ Briefly, cognitive testing was conducted by trained staff at baseline, 1 year, and every 2 years thereafter until the end of the clinical trial. Participants meeting predefined “triggers” (e.g., low (<78) or declining (drop of more than 10.15 points below predicted) 3MS scores, specialist concern, or documented diagnosis/treatment) underwent further standardized cognitive and functional assessments. A diagnosis of dementia was confirmed by an adjudication committee using Diagnostic and Statistical Manual of Mental Disorders, Fourth Edition (DSM‐IV) criteria and dated to the initial trigger leading to adjudication.^12^ Major bleeding was defined as clinically significant bleeding, hemorrhagic stroke, or subarachnoid hemorrhage.^9^

### 2.3 Genotyping and polygenic scores

Genotyping was carried out on 14,117 baseline DNA samples using the Axiom 2.0 Precision Medicine Diversity Array (Thermo Fisher Scientific, CA). Variant calling of ∼850,000 variants was done using a customized pipeline, aligning data to the human reference genome GRCh38 (hg38). Low‐quality samples and single nucleotide polymorphisms (SNPs) were removed during quality control (QC) steps,^13^ leaving 13,571 participants. Genetic ancestry and relationships were inferred^14^ and included 96.6% of European decent. Imputation, increasing SNP data to > 19 million datapoints, was performed using the TOPMed Imputation Server with the TOPMed “r3” reference panel, containing 133,597 genetically diverse reference samples with data from over 445 million genetic variants.^15^, ^16^, ^17^ Variants were removed in post‐imputation QC if imputation quality score *R* ^2^ was < 0.3. Genetic principal components were derived from genome‐wide genotype data and summarize major axes of genetic variation related to ancestral diversity within the cohort. These variables are commonly included in genetic association studies to reduce bias from ancestry‐related differences in allele frequencies. *APOE* genotype was measured directly at two SNPs, rs7412 and rs429358, which were included in the Axiom array, with available genotypes for 13,541 participants that were used for further analysis (Figure S1).

We downloaded 5182 PGSs from PGS Catalog (on October 28, 2025).^8^, ^18^ Each PGS was calculated on the 13,541 genotyped ASPREE participants using PLINK 1.9.^19^ Then, each PGS itself underwent QC in two steps. First, we removed any PGS that did not have >95% SNPs available in the ASPREE genotyped dataset. Second, PGSs published prior to 2021 were excluded to focus the analysis on contemporary scores developed using more recent genomic wide association study (GWAS) resources and PGS methodologies. This criterion was applied after score‐level QC and resulted in the exclusion of a small number of additional scores (*n* = 62). Third, GWASs that did not use European samples to derive the score were also removed. Technical QC was then carried out, based on the central limit theorem, to assure an approximately normal distribution of each PGS.^20^ This included removing PGSs with a multimodal distribution,^21^ high kurtosis (long tails in distribution, i.e., >5), and over 1% of their data outside of 3 standard deviations (SD). After this QC, 1848 PGS remained for further testing.

### 2.4 Statistical analysis

For each PGS, Cox proportional‐hazards models were fitted with dementia as the outcome, including terms for PGS, aspirin allocation, and their interaction, adjusted for age, sex, number of *APOE* ε3 and *APOE* ε4 alleles, and the first 10 principal components, included to account for subtle differences in genetic ancestry within the cohort and to minimize confounding arising from population stratification. Interaction *p* values were Bonferroni‐corrected for multiplicity across 1848 tested PGSs (*α* = 0.05/1848). For PGSs showing significant interactions, restricted cubic spline functions were used to visualize the continuous relationship between the PGS and the hazard ratio (HR) for aspirin versus placebo, allowing for potential non‐linear associations across the PGS distribution. PGSs showing significant interactions were further examined in quintiles, comparing aspirin and placebo groups within each quintile, with placebo as the reference.

Models first included the above adjustments, as well as models further adjusting for baseline smoking status, alcohol consumption, body mass index, hypertension, diabetes, 3MS score and education level (≤12 years vs ≥13 years), and prior use of aspirin or anti‐inflammatory drugs. Bleeding outcomes were also assessed using Cox models and screen‐wide significant PGSs. To assess whether all‐cause mortality could compete with dementia estimates, we conducted competing risk sensitivity analyses using Fine–Gray subdistribution hazards models treating death as a competing event. All analyses were conducted in R version 4.5.1.

## 3 RESULTS

### 3.1 Participant characteristics

Baseline characteristics, dementia, and bleeding endpoints of all participants are presented in Table 1. Of the 13,541 participants, 345 were diagnosed with dementia over a median follow‐up of 4.6 years (interquartile range [IQR]: 3.55 to 5.62). Mean age was 75 years (SD 4.3), and 54.8% were female. Most participants had hypertension, < 13 years education, had never smoked, and were current drinkers.

**TABLE 1:** Participant baseline characteristics and trail period endpoints.

Baseline characteristics	All participants (*n* = 13,541)
Age, mean (SD), years	75 (4.3)
Sex female, *n* (%)	7421 (54.8)
BMI (kg/m^2^), mean (SD)	28 (4.6)
Hypertension, *n* (%)	9969 (73.6)
Diabetes, *n* (%)	1318 (9.7)
Baseline 3MS, mean (SD)	93.7 (4.4)
Education ≥13 years, *n* (%)	5778 (42.7)
Smoking status, *n* (%)	
Current/former	6009 (44.4)
Never	7532 (55.6)
Alcohol use, *n* (%)	
Current	10,680 (78.9)
Former/never	2861 (21.1)
Medication	
Statins, *n* (%)	4174 (30.8)
NSAID, *n* (%)	1791 (13.2)
Previous aspirin use, *n* (%)	1295 (9.6)
Trail aspirin allocation, *n* (%)	6742 (49.8)
Cholesterol	
HDL (mmol/L), mean (SD)	1.58 (0.46)
LDL (mmol/L), mean (SD)	3.07 (0.87)
*APOE* genotype	
ε1/ε3: ε2/ε4, *n* (%)	485 (3.6)
ε1/ε4, *n* (%)	2 (0.01)
ε2/ε2, *n* (%)	74 (0.6)
ε2/ε3, *n* (%)	1852 (13.7)
ε3/ε3, *n* (%)	8140 (60)
ε3/ε4, *n* (%)	2778 (20.5)
ε4/ε4, *n* (%)	210 (1.6)
Endpoints, *n* (%)	
Dementia	345 (2.5)
Major bleeding	414 (3.1)

Abbreviations: BMI, body mass index; HDL, high‐density lipoprotein; LDL, low‐density lipoprotein; NSAID, non‐steroidal anti‐inflammatory drug use.

### 3.2 Polygenic score screening

Of the 1848 PGSs that passed QC, 112 showed a significant interaction with aspirin. After adjusting for Bonferroni correction, three PGSs for “mean platelet (thrombocyte) volume” passed multiple testing, PGS002189 (*p* = 3.48E‐06, Bonferroni *p* = 0.0046), PGS003548 (*p* = 1.18E‐05, Bonferroni *p* = 0.022), and PGS001971 (*p* = 1.51E‐05, Bonferroni *p* = 0.028). Results for all 1848 quality‐controlled PGSs in basic Cox models with PGS × aspirin interaction *p* values can be seen in File S1. PGS001971 and PGS002189 were from the same study but using different methods of generation and number of variants (65,450 and 462,934 variants from 391,124 individuals, respectively),^22^ and PGS003548 was from a separate study (979,739 variants from 359,833 individuals).^23^

These three PGSs were highly correlated to each other (Pearson correlation *r* > 0.94) and therefore likely represent a single platelet‐related genetic signal. PGS003548 was selected as a representative score for the primary analyses because it incorporated the largest number of variants and produced interaction estimates comparable to the other top‐ranked platelet‐related scores. Similar results were observed for the other Bonferroni‐significant platelet‐count PGSs (PGS002189 and PGS001971), indicating that the findings were not dependent on the choice of one specific score.

Results for all follow‐up analyses on PGS001971 and PGS002189 are presented in parallel in File S2. The continuous interactions between PGS003548, PGS001971, and PGS002189 and aspirin allocation are illustrated using spline curves for dementia and bleeding outcomes (File S2 and Figures S2–S7). Although the interaction was significant between PGS003548 × Apsirin (*p* = 1.18×10^−5^, Bonferroni *p* = 0.022), neither the continuous PGS003548 score nor randomization to aspirin in the continuous Cox model were independently associated with incident dementia (HR: 1.13, *p* = 0.10 and HR: 0.85, *p* = 0.14, respectively).

### 3.3 *APOE* and dementia‐related genetic benchmark analyses

As a benchmark analysis, we assessed whether *APOE* genotype modified the effect of aspirin on dementia risk. While *APOE* genotype was strongly associated with dementia risk, there was no evidence of *APOE* × aspirin interaction (all interaction *p* values > 0.59; File S2 and Table S1).

We additionally examined dementia‐related PGSs within the interaction screen. Among the 1848 PGSs evaluated, 18 related to dementia, Alzheimer's disease, or Parkinson's disease. None demonstrated evidence of interaction with aspirin after correction for multiple testing. One score (PGS004863) showed a nominal interaction (*p* = 0.03), but this did not survive adjustment for multiple comparisons.

### 3.4 Sensitivity exploration of PGSs screened

Platelet‐related polygenic scores were markedly overrepresented among nominally significant dementia–aspirin interaction signals. To determine whether the overrepresentation of platelet‐related PGSs among the strongest aspirin–dementia interaction signals exceeded that expected from their frequency in the tested set, all 1848 QC‐passing PGS were classified into broad biological trait domains (File S2 and Table S2). Twenty‐four PGSs (1.3%) were classified as platelet‐related traits. Despite this, platelet‐related PGSs accounted for 18 of 112 nominally significant interaction results, compared with only 1.45 expected by chance (enrichment ratio = 12.4). This corresponded to a 54.7‐fold increase in the odds of significance relative to non‐platelet PGS (Fisher's exact *p* = 3.1 × 10^−^^1^ ^8^; false discovery rate [FDR] = 6.5 × 10^−^^1^ ^7^).

In contrast, no other biological trait category demonstrated comparable enrichment among the nominally significant interaction signals. Although PGSs for cancer traits showed modest enrichment among the nominally significant interaction results (odds ratio [OR] = 2.67, FDR = 0.0036), none passed multiple testing, and platelet‐related PGSs exhibited substantially stronger enrichment than any other biological domain.

Pairwise correlation analyses of the 18 nominally significant platelet‐related PGSs demonstrated substantial heterogeneity in correlation structure (Figure S8). As expected, the three Bonferroni‐significant PGSs formed a highly correlated cluster (*r* = 0.94 to 0.97), consistent with these scores representing closely related platelet‐count genetic signals. However, other platelet‐related PGSs showed considerably weaker correlations, indicating that the platelet enrichment signal was not solely attributable to a single PGS cluster but instead reflected multiple related platelet‐count genetic signals.

### 3.5 Genetic stratification of PGS003548

Results from the fully adjusted analysis of aspirin versus placebo within PGS003548 quintile strata can be seen in Table 2 (simple adjustment in Table S3, File S2, full model for PGS001971 and PGS002189, Table S4). The platelet PGS reflects genetically predicted platelet count, ranging from the lowest (Q1) to highest (Q5) quintile. We observed a potential aspirin effect pattern with the increase of PGS, although this pattern was not significant for Q1 to 4. However, the overall pattern was consistent with a possible crossing interaction in which the effect of aspirin differed according to platelet genetic risk. Thus, we cannot rule out the possibility of harm for aspirin in lower‐PGS strata.

**TABLE 2:** Risk of dementia and major bleeding in aspirin versus placebo, stratified by quintiles of a platelet polygenic score.

		Dementia				Major Bleeding			
PGS ID	Quintile	Events	HR	95%CI	*p* value^*^	Events	HR	95%CI	*p* value^*^
PGS003548	Q1	76	1.36	0.85, 2.20	0.20	72	0.95	0.59, 1.51	0.82
	Q2	75	1.09	0.69, 1.72	0.72	85	1.30	0.85, 1.99	0.23
	Q3	62	0.96	0.58, 1.59	0.87	92	1.25	0.83, 1.88	0.29
	Q4	73	0.92	0.57, 1.48	0.72	78	1.07	0.68, 1.68	0.76
	Q5	59	0.29	0.16, 0.55	0.00012	87	2.08	1.33, 3.26	0.0013

*Note*:^*^Adjusted for age, sex, baseline smoking status, alcohol consumption, body mass index, hypertension, diabetes, 3MS score and education level (≤12 years vs ≥13 years), *APOE* ε2 allele count and *APOE* ε4 allele count, prior use of aspirin or anti‐inflammatory drugs, and top 10 genetic principal components.

**FIGURE 1:**
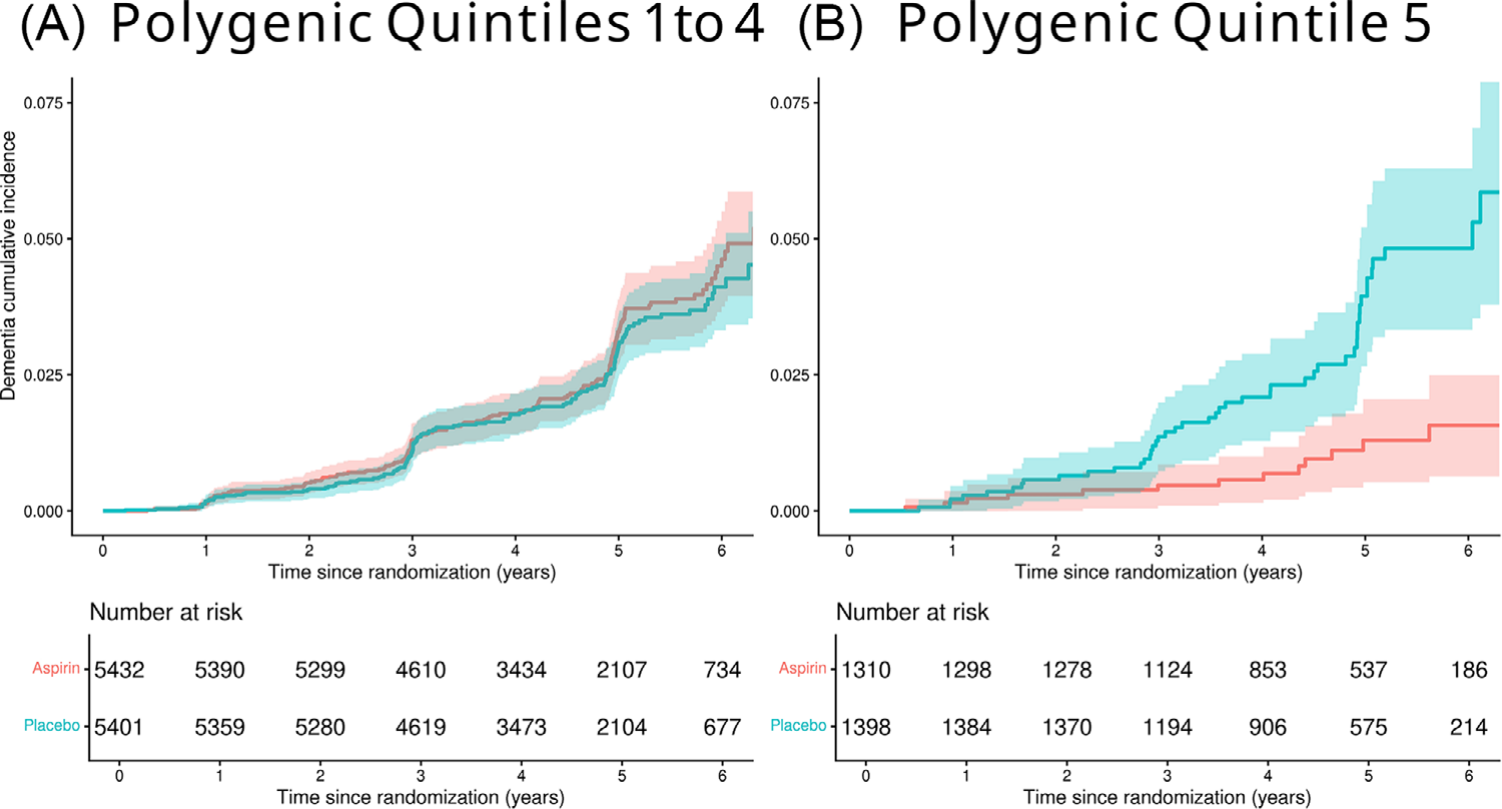
Dementia cumulative incidence stratified by a platelet count polygenic score. (A) Cumulative incidence for 6‐year dementia in PGS003548 quintiles 1 to 4: *n* = 149 dementia endpoints in aspirin allocation group, *n* = 137 dementia endpoints in placebo allocation group. (B) Cumulative incidence for 6‐year dementia, PGS003548 quintile 5: *n* = 13 dementia endpoints in aspirin allocation group, *n* = 46 dementia endpoints in placebo allocation group.

Participants randomized to aspirin in Q5 of the PGS distribution (the highest quintile for genetically associated platelet count) showed marked reduction in dementia risk specifically, with aspirin use being associated with 3.5‐fold lower dementia risk versus placebo (HR: 0.29, 95% confidence interval [CI]: 0.16 to 0.55, *p* = 0.0001). This association remained relatively unchanged after accounting for the competing risk of death using Fine–Gray subdistribution hazard models (sHR: 0.31, 95% CI: 0.16 to 0.57, *p* = 0.0005). Cumulative incidences of dementia, comparing aspirin to placebo in Q1 to Q4 and Q5 quintile groups, are shown in Figure 1. Cumulative incidences of bleeding for PGS003548, and dementia and bleeding for PGS001971 and PGS002189, are shown in File S2 and Figure S9, S10, and S11, respectively.

In Q5 of the PGS distribution, an increased risk of major bleeding events was also observed when comparing participants randomized to aspirin versus placebo (HR: 2.08, 95% CI: 1.33 to 3.26, *p* = 0.001).

Comparisons of baseline characteristics and study endpoints between placebo‐ and aspirin‐allocated groups within Q5 can be seen in Table 3 (PGS001971 and PGS002189; File S2 and Table S5). There were no significant differences between the two groups (all *p* > 0.05), aside from dementia and major bleeding endpoints. Dementia and major bleeding events in each quintile and incidence per 1000 person‐years stratified by treatment group can be seen in Table 4.

**TABLE 3:** Comparison of baseline characteristics and endpoints between aspirin versus placebo allocated participants within polygenic score Quintile 5.

	PGS003548 (Quintile 5) (*n* = 2708)		
Baseline characteristics	Aspirin (*n* = 1310)	Placebo (*n* = 1398)	*p* value
Age, mean (SD)	75.1 (4.5)	74.9 (4.3)	0.15
Sex female, *n* (%)	722 (55.1)	761 (54.5)	0.72
BMI (kg/m^2^), mean (SD)	28.3 (4.5)	28.1 (4.7)	0.38
Hypertension, *n* (%)	977 (74.4)	1038 (74.3)	0.88
Diabetes, *n* (%)	126 (9.6)	145 (10.4)	0.56
Baseline 3MS, mean (SD)	93.7 (4.4)	93.8 (4.3)	0.70
Education ≥13 years, *n* (%)	533 (59)	598 (42.8)	0.28
Smoking status, *n* (%)			
Current/former	585 (44.7)	632 (45.2)	0.80
Never	725 (55.4)	766 (54.8)	
Alcohol use, *n* (%)			
Current	1037 (79.1)	1119 (80)	0.60
Former/never	273 (20.9)	279 (20)	
Medication			
Statins, *n* (%)	407 (31.1)	412 (29.4)	0.38
NSAID, *n* (%)	167 (12.8)	193 (13.9)	0.45
Previous aspirin use, *n* (%)	114 (8.8)	122 (8.7)	0.99
Cholesterol			
HDL (mmol/L), mean (SD)	1.57 (0.45)	1.57 (0.44)	0.74
LDL (mmol/L), mean (SD)	3.05 (0.86)	3.07 (0.86)	0.58
*APOE* genotype			
ε1/ε3: ε2/ε4, *n* (%)	39 (3)	45 (3.2)	0.94
ε1/ε4, *n* (%)	0 (0)	0 (0)	
ε2/ε2, *n* (%)	10 (0.8)	7 (0.5)	
ε2/ε3, *n* (%)	182 (13.9)	203 (14.4)	
ε3/ε3, *n* (%)	796 (60.6)	852 (60.9)	
ε3/ε4, *n* (%)	268 (20.4)	275 (19.6)	
ε4/ε4, *n* (%)	15 (1.1)	16 (1.1)	
Endpoints, *n* (%)			
Dementia	13 (0.99)	46 (3.3)	<0.0001
Major bleeding	58 (4.4)	29 (2.1)	0.0008

Abbreviations: BMI, body mass index; HDL, high‐density lipoprotein; LDL, low‐density lipoprotein; NSAID, non‐steroidal anti‐inflammatory drug use.

**TABLE 4:** Dementia and major bleeding events during the ASPREE trial, stratified by PGS003548 polygenic risk quintiles in aspirin and placebo groups separately.

			No. events for dementia					No. events for major bleeding				
	No. participants		Placebo		Aspirin		Dementia cases averted	Placebo		Aspirin		Bleeding events induced
Polygenic risk quintiles	Placebo	Aspirin	Total	Per 1000 person‐years	Total	Per 1000 person‐years	Per 1000 person‐years	Total	Per 1000 person‐years	Total	Per 1000 person‐years	Per 1000 person‐years
Quintile 1	1347	1362	29	4.80	47	7.62	−2.82	35	5.83	37	5.99	0.16
Quintile 2	1371	1337	36	5.82	39	6.53	−0.71	38	6.15	47	7.89	1.74
Quintile 3	1352	1356	32	5.20	30	4.89	0.31	42	6.85	50	8.23	1.38
Quintile 4	1331	1377	40	6.60	33	5.29	1.31	37	6.12	41	6.61	0.49
Quintile 5	1398	1310	46	7.18	13	2.18	5	29	4.51	58	9.81	5.3
Overall	6799	6742	183	5.93	162	5.32	0.61	181	5.88	233	7.69	1.81

To determine whether the interaction signals identified by the Bonferroni‐significant platelet PGS reflected a broader platelet‐related genetic signal rather than isolated findings, we examined aspirin‐associated treatment effects across all 24 platelet‐related PGS that passed QC. The strongest dementia‐protective aspirin effect estimates were concentrated among the platelet‐count PGS that ranked highest in the interaction screen, including the three Bonferroni‐significant scores (PGS003548, PGS002189, and PGS001971) (File S2 and Figure S12). In contrast, platelet‐related scores that did not emerge as interaction signals generally demonstrated weaker and less consistent treatment effects. Examination of major bleeding outcomes showed that increased bleeding risk with aspirin was observed across many platelet‐related PGSs, with most treatment effect estimates in the direction of increased bleeding risk (File S2 and Figure S13).

Across all 24 platelet‐related PGSs, 13 (54.2%) demonstrated the coherent pattern of aspirin‐associated dementia protection together with increased major bleeding risk, while 19 (79.2%) showed treatment effect estimates in the direction of increased bleeding risk. Median treatment effect estimates across platelet‐related PGSs were also directionally consistent with reduced dementia risk (median HR = 0.84) and increased major bleeding risk (median HR = 1.27). The relationship between aspirin‐associated dementia and major bleeding treatment coefficients across all 24 platelet‐related PGSs is shown in File S2 and Figure S14. The numerical results underlying these analyses are provided in File S3.

Stratification sensitivity analyses using alternative genetic risk thresholds produced consistent results across all three Bonferroni‐significant platelet‐related PGSs (PGS003548, PGS002189, and PGS001971) (Table S6). For PGS003548, aspirin‐associated dementia risk reduction was observed in the highest tertile (HR = 0.48, 95% CI: 0.32 to 0.72), highest decile (HR = 0.09, 95% CI: 0.02 to 0.41), and the highest 5% of the distribution (HR = 0.07, 95% CI: 0.008 to 0.58). All results were statistically significant. Similar patterns were observed for PGS002189 and PGS001971 (Table S6). The progressively stronger associations observed in increasingly extreme genetic risk strata are consistent with the possibility that the aspirin–dementia interaction is most strongly concentrated among individuals at the upper end of the platelet genetic risk distribution.

## 4 DISCUSSION

To our knowledge, this is the first study to report a genetic subgroup of individuals in whom low‐dose aspirin may reduce the incidence of dementia, albeit with increased bleeding risk. We developed a novel screening method, quality controlling and testing over 5000 publicly available PGSs to identify genetic subgroups who may benefit in this way, and our hypothesis‐generating study identified an enrichment of potential benefit on aspirin for a specific subgroup, based on PGSs related to platelet count. We further examined three overlapping platelet count PGSs that demonstrated robust, Bonferroni‐significant modification of aspirin treatment effects. These PGSs demonstrated a potential to identify genetic subgroups of older individuals who may derive preventive benefit from aspirin for dementia, thereby suggesting a more targeted role for a medication previously considered ineffective in primary prevention. However, we acknowledge the exploratory and hypothesis‐generating nature of our investigation.

Although point estimates in several lower quintiles were directionally consistent with a neutral to harmful effect of aspirin, these comparisons were not statistically significant, potentially due to the small sample size. Therefore, these observations should be interpreted cautiously. For participants in the highest quintile of the platelet PGS, aspirin allocation was associated with a substantial 3.5‐fold risk reduction in incident dementia versus placebo.

From a clinical perspective, the observed reduction in dementia risk in the highest platelet PGS quintile must be weighed against the concurrent increase in major bleeding risk. Based on observed event rates, the absolute reduction in dementia risk was approximately 2.3% (3.3% vs 1.0%), corresponding to a number needed to treat of approximately 44, while the absolute increase in major bleeding risk was approximately 2.3% (2.1% vs 4.4%), corresponding to a number needed to harm of again approximately 44. Expressed differently, aspirin was associated with approximately five fewer dementia cases but five additional major bleeding events per 1000 person‐years in this Q5 subgroup. These findings highlight the complexity of balancing potential dementia prevention benefits against bleeding harms in this age group and underscore the importance of careful benefit–harm assessment before considering any clinical implementation. Importantly, no difference in all‐cause mortality was observed between aspirin and placebo in the Q5 subgroup, suggesting that the observed benefits and harms did not translate into an overall survival difference.

Notably, we did not observe significant aspirin interaction effects for *APOE* genotype or for dementia and Alzheimer's disease‐related PGSs. Given that these are among the most well‐established genetic predictors of dementia risk, this finding suggests that the aspirin interaction identified in this study reflects platelet biology pathways rather than genetic susceptibility to dementia per se.

Thus, our findings raise the possibility that aspirin's benefit for dementia prevention may be altered by underlying genetics, specifically with regard to platelet count. Platelet count is a highly heritable trait (30% to 80%), and genetic variation influencing platelet count has been associated with multiple health outcomes, although previous studies did not report associations with dementia or bleeding.^24^, ^25^, ^26^

Most evidence of a link between platelets and dementia center around platelet function or aggregation rather than count. The platelet PGSs identified in our study were derived from platelet‐count GWASs rather than direct measures of platelet activation or aggregation. Therefore, caution is warranted when linking our findings to prior studies of platelet reactivity, activation, aggregation, and dementia. Platelet reactivity, activation, and aggregation are biologically distinct phenotypes only partially overlapping with platelet count.

Nevertheless, platelet count is a clinically relevant phenotype and has been associated with cerebrovascular pathology, including white matter hyperintensities.^27^, ^28^ More broadly, platelets are increasingly recognized as contributors to inflammatory and cardiovascular processes,^26^, ^29^, ^30^ suggesting that the identified PGSs may capture aspects of platelet‐related biology relevant to vascular aging. However, the specific biological mechanism underlying the observed interaction remains uncertain.

The enrichment of several platelet count–related scores among the highest‐ranked interaction signals supports the interpretation that Bonferroni‐significant scores were not entirely isolated. The marked enrichment of many platelet‐related PGSs among the significant interaction signals shows consistency of platelet‐related genetic variation modifying aspirin's effect on dementia. However, these observations should not be interpreted as independent replication because many platelet‐related scores were highly correlated.

Participants allocated to placebo within the highest platelet PGS quintile experienced a higher incidence of dementia than the overall cohort. This raises the possibility that high genetically predicted platelet count may itself be associated with increased dementia risk, independent of aspirin exposure. However, we did not observe a significant association between the continuous platelet PGS and dementia risk in the overall cohort or placebo arm, suggesting that any relationship may not be linear across the full distribution of genetically predicted platelet count. Instead, risk may be concentrated among individuals at the extreme upper end of the distribution, consistent with a potential threshold effect.

Cumulative incidence curves for dementia in the highest platelet–PGS quintile began to diverge primarily after approximately 2 to 3 years of follow‐up. Although this pattern may reflect a meaningful biological effect, the modest number of dementia events limits confidence in its interpretation. However, it is consistent with the possibility that any beneficial effect of aspirin on dementia risk requires prolonged exposure before becoming clinically apparent. Alternatively, the delayed separation may reflect the timing of dementia ascertainment in ASPREE, which relied on periodic cognitive assessments and adjudicated diagnoses rather than continuous surveillance.

### 4.1 Strengths and limitations

The strengths of this study are the randomized ASPREE trial cohort, one of the largest placebo‐controlled trials of aspirin in healthy older adults, with genotypic data and rigorous ascertainment and adjudication of dementia and bleeding outcomes. Our study employed a comprehensive, unbiased screen of eligible PGSs from the PGS Catalog using stringent QC procedures and Bonferroni correction for multiple testing. Consistent findings observed across alternative risk thresholds and different, highly correlated platelet‐count PGSs support the stability of the observed signal. The availability of both dementia and major bleeding endpoints within the same cohort enabled simultaneous evaluation of potential therapeutic benefits and harms, which is essential for assessing aspirin's net clinical utility in any subgroup of interest.

The limitations of the study include the exploratory, post hoc nature of the analysis, making the findings more hypothesis‐generating and therefore still requiring independent replication. A further limitation is the absence of external validation. The identified platelet PGS interactions were identified within the same cohort and therefore may reflect cohort‐specific characteristics, despite the application of QC procedures and Bonferroni correction. Independent replication in external cohorts and clinical studies would strengthen the findings. However, we acknowledge that replication of these findings will be challenging given that, to our knowledge, ASPREE is the only large, randomized placebo‐controlled aspirin primary prevention trial with dementia outcomes and genotyping. Thus, we believe that no directly comparable external dataset is currently available for validation.

Our analysis derived from a screen‐wide search of 1848 PGS, so the magnitude of the observed effect should be interpreted cautiously. Selection of the strongest interaction signal may inflate the estimated effect size, and replication will be required to establish the true magnitude of the association. Further, the follow‐up period was relatively short (4.7 years) when considering the gradual onset of dementia. Thus, the study may have preferentially captured individuals with more rapidly progressing or clinically apparent disease, while participants with longer preclinical phases may not have reached the dementia trial endpoint. Dementia events within each quintile were limited and consequently some confidence intervals were wide. Dementia subtype analyses were not performed because further subdivision of cases according to treatment allocation, genetic risk strata, and clinical subtype would have resulted in limited statistical power. Thus, analyses were restricted to all‐cause dementia.

Measured platelet phenotypes such as platelet count, aggregation, and activation were not available in the ASPREE trial, limiting the scope of follow‐up analyses. In addition, the PGS Catalog currently includes scores for platelet count and size but not platelet aggregation, activation, or response, limiting the ability to further interrogate underlying genetic mechanisms related to these properties with other PGS.

The platelet PGSs evaluated in this study were derived from platelet‐count GWASs conducted in populations that differed from ASPREE in age. Relationships between platelet count, function, and reactivity may change with aging, and platelet count may not fully capture biologically relevant variation in platelet activation among older adults. Future studies incorporating genetic predictors of platelet function or direct measures of platelet reactivity will be important for clarifying the mechanisms underlying the observed interaction. Finally, the predominantly European ancestry of the ASPREE cohort restricts the generalizability of this study's findings to other ancestry populations.

## 5 CONCLUSION

A high PGS related to platelet count may identify a subgroup of individuals whose dementia risk is substantially reduced by low‐dose aspirin. These findings highlight the potential for genetically informed, risk–benefit assessment of aspirin in the primary prevention setting for dementia. Our findings raise the possibility that future prevention strategies for dementia could be more precision‐guided and targeted to defined subgroups based on genetics. However, the findings of this exploratory and hypothesis‐generating study require further external validation, interpretation, and investigation.

## AUTHOR CONTRIBUTIONS

Peter D. Fransquet conceptualized the study. Peter D. Fransquet undertook data analysis, interpretation, and writing of the first draft manuscript with the input of Paul Lacaze and Chenglong Yu. All authors, including Peter D. Fransquet, Chenglong Yu, Cammie Tran, Sultana Monira Hussain, Amy Brodtmann, Andrew M. Tonkin, Mark R Nelson, Joanne Ryan, John J. McNeil, and Paul Lacaze, contributed to the writing, direction, and editing of the final draft. All authors read and approved the final manuscript.

## CONFLICT OF INTEREST STATEMENT

There are no conflicts of interest to disclose for any authors of this work. Author disclosures are available in the Supporting Information.

## CONSENT STATEMENT

All participants of the ASPREE study gave informed written consent.

## Supporting information



Supporting Information

Supporting Information

Supporting Information

Supporting Information

## Data Availability

Due to the nature of genomics, the de‐identified data we analyzed are not publicly available, but requests to the corresponding author for the data will be considered on a case‐by‐case basis. The complete ranked results from the screen, including all 1848 PGS × aspirin interaction statistics, have been provided in File S1.
