# Supplementary material for: Platelet count polygenic scores may modify the effect of aspirin in primary prevention of dementia
Source: Alzheimers Dement. 2026 Sep 24;22(9):e71817. doi: 10.1002/alz.71817 (PMC13602355; doi:10.1002/alz.71817)
Supplement: Supplementary file 2 [file ALZ-22-e71817-s002.docx]

**SUPPLEMENTART FILE 2 for: Platelet count polygenic scores may modify the effect of aspirin in primary prevention of dementia**

**Results**

**Continuous PGS results for PGS001971**

The interaction between **PGS001971**xApsirin xApsirin was significant (p=1.51x10^-5^, Bonferroni p = 0.028), neither the continuous PGS001971 score nor randomisation to aspirin in the continuous Cox model were independently associated with incident dementia (HR:1.14, p=0.08 and HR:0.86, p=0.16, respectively). Spline curves for the continuous interaction between PGS001971 and aspirin allocation for dementia and major bleeding can be seen in figure S2 and S3 respectively.

**Genetic Stratification for PGS001971**

For participants in the highest quintile (Q5) of the PGS001971 distribution, there were marked differences in dementia risk between aspirin and placebo groups (Table S3). Individuals who were assigned aspirin in Q5 had a 2.3-fold lower risk of dementia (HR:0.43, 95%CI: 0.24 - 0.77, p-value = 0.0044) versus placebo. However, individuals in Q5 randomized to aspirin also had an increased risk of major bleeding versus placebo (HR:1.94, 95%CI: 1.27-2.97, p-value = 0.0022). Observations remained significant in fully adjusted models apart from the Q4 observation (Table S4). Cumulative incidence of dementia and bleeding, comparing aspirin to placebo in stratified quintile groups (Q1-4 and Q5) can been seen in Figure S10.

**Continuous PGS results for PGS002189**

The interaction between PGS002189xApsirin was significant (p=2.48x10^-6^, Bonferroni p = 0.0046), neither the continuous PGS002189 score nor randomisation to aspirin in the continuous Cox model were independently associated with incident dementia (HR:1.14, p=0.09 and HR:0.84, p=0.12, respectively). Spline curves for the continuous interaction between PGS002189 and aspirin allocation for dementia and major bleeding can be seen in figure S4 and S5 respectively.

**Genetic Stratification for PGS002189**

Participants in Q1 of the PGS002189 distribution showed an increase in risk of dementia with aspirin allocation (HR:1.62, 95%CI: 1.02 - 2.59, p-value = 0.04). For participants in the highest quintile (Q5) of the PGS002189 distribution, there were marked differences in dementia risk between aspirin and placebo groups (Table S1). Individuals who were assigned aspirin in Q5 had a 3.3-fold lower risk of dementia (HR:0.30, 95%CI: 0.16 - 0.57, p-value = 0.00022) versus placebo. However, individuals in Q5 randomized to aspirin also had an increased risk of major bleeding versus placebo (HR:1.97, 95%CI: 1.27-3.07, p-value = 0.0027). Bleeding risk was also elevated with aspirin in Q4 of the PGS002189 distribution (HR:1.67, 95%CI: 1.05 - 2.68, p-value = 0.031). Observations remained significant in fully adjusted models apart from the Q1 observation for dementia (Table S4). Cumulative incidence of dementia and bleeding, comparing aspirin to placebo in stratified quintile groups (Q1-4 and Q5) can been seen in Figure S11.

**Figure S1. Flow chart for study sample selection.**

 **Figure S2. Spline curve for the continuous interaction between PGS001971 and aspirin allocation for dementia.** Natural cubic spline functions were specified with boundary knots at the 5th and 95th percentiles and interior knots at the 35th and 65th percentiles of the PGS distribution.

**Figure S3. Spline curve for the continuous interaction between PGS001971 and aspirin allocation for bleeding.** Natural cubic spline functions were specified with boundary knots at the 5th and 95th percentiles and interior knots at the 35th and 65th percentiles of the PGS distribution.

**Figure S4. Spline curve for the continuous interaction between PGS002189 and aspirin allocation for dementia.** Natural cubic spline functions were specified with boundary knots at the 5th and 95th percentiles and interior knots at the 35th and 65th percentiles of the PGS distribution.

**Figure S5. Spline curve for the continuous interaction between PGS002189 and aspirin allocation for bleeding.** Natural cubic spline functions were specified with boundary knots at the 5th and 95th percentiles and interior knots at the 35th and 65th percentiles of the PGS distribution.

**Figure S6. Spline curve for the continuous interaction between PGS003548 and aspirin allocation for dementia.** Natural cubic spline functions were specified with boundary knots at the 5th and 95th percentiles and interior knots at the 35th and 65th percentiles of the PGS distribution.

**Figure S7. Spline curve for the continuous interaction between PGS003548 and aspirin allocation for major bleeding.** Natural cubic spline functions were specified with boundary knots at the 5th and 95th percentiles and interior knots at the 35th and 65th percentiles of the PGS distribution.

**Figure S8. Correlation Heatmap of Nominally Significant Platelet Count Polygenic Scores.**

**Figure S9. Bleeding 6-year Cumulative Incidence for PGS003548**

a) Cumulative incidence for 6-year dementia, platelet PGS003548 quintiles 1-4

b) Cumulative incidence for 6-year dementia, platelet PGS003548 quintile 5

**Figure S10. Dementia and Bleeding 6 year Cumulative Incidence for PGS001971**

a) Cumulative incidence for 6-year dementia, platelet PGS001971 quintiles 1-4

b) Cumulative incidence for 6-year dementia, platelet PGS001971 quintile 5

c) Cumulative incidence for 6-year bleeding, platelet PGS001971 quintiles 1-4

d) Cumulative incidence for 6-year bleeding, platelet PGS001971 quintiles 5

**Figure S11. Dementia and Bleeding 6 year Cumulative Incidence for PGS002189**

a) Cumulative incidence for 6-year dementia, platelet PGS002189 quintiles 1-4

b) Cumulative incidence for 6-year dementia, platelet PGS002189 quintile 5

c) Cumulative incidence for 6-year bleeding, platelet PGS002189 quintiles 1-4

d) Cumulative incidence for 6-year bleeding, platelet PGS002189 quintiles 5

**Figure S12. Forest plot of highest genetic risk quintile (Q5) of each platelet-related polygenic score (PGS) for Aspirin vs Placebo on Dementia Outcome.** Ranges indicate 95% confidence intervals. Vertical dashed line indicates no treatment effect (HR = 1.0). Estimates were derived from Cox proportional hazards models adjusted for age, sex, APOE genotype, and top 10 genetic principal components.

**Figure S13. Forest plot of highest genetic risk quintile (Q5) of each platelet-related polygenic score (PGS) for Aspirin vs Placebo on Major Bleeding outcome.** Ranges indicate 95% confidence intervals. Vertical dashed line indicates no treatment effect (HR = 1.0). Estimates were derived from Cox proportional hazards models adjusted for age, sex, APOE genotype, and top 10 genetic principal components.

**Figure S14. Relationship between aspirin treatment coefficients for dementia and major bleeding across all 24 platelet-related polygenic scores (PGS) in the highest genetic risk quintile (Q5).** Dashed vertical and horizontal lines indicate no treatment effect (β = 0). Points in the upper-left quadrant represent the hypothesised coherent pattern of aspirin-associated dementia protection (β < 0) together with increased major bleeding risk (β > 0).

**Table S1. APOExAspirin interaction analysis**

| **APOE Variable** | **Comparison** | **Main HR** | **Main 95%CI** | **Main P-val** |  | **Int HR** | **Int 95%CI** | **Int P-val** |
| --- | --- | --- | --- | --- | --- | --- | --- | --- |
| **APOE ε4** | 1 allele vs 0 | 2.7 | 2.00 - 3.65 | 9.57E-11 |  | 0.9 | 0.58 – 1.40 | 0.637 |
|  | 2 alleles vs 0 | 6.16 | 3.19 - 11.9 | 6.43E-08 |  | 0.77 | 0.29 – 2.03 | 0.593 |
| **APOE ε2** | 1 allele vs 0 | 0.59 | 0.37 - 0.93 | 0.0239 |  | 1.01 | 0.52 – 1.98 | 0.97 |
|  | 2 alleles vs 0 | 0 | 0 - Inf | 0.992 |  | 1.15 | 0 - Inf | >0.99 |

CI, confidence interval; HR, hazard ratio; int, interaction.

Estimates for individuals carrying two APOE ε2 alleles should be interpreted cautiously due to the small number/no participants and dementia events in this subgroup.

**Table S2. Sensitivity exploration of 1,848 QC-passing PGS, classified into broad biological trait domains^#^ (nominally signification PGS n=112)**

| **PGS Trait Group** | **Group Total** | **Sig* in Group** | **Expected in Total** | **Enrichment ratio** | **Odds ratio** | **P value** | **FDR** |
| --- | --- | --- | --- | --- | --- | --- | --- |
| **Platelet** | 24 | 18 | 1.45 | 12.38 | 54.74 | 3.11E-18 | 6.54E-17 |
| **Cancer** | 159 | 21 | 9.64 | 2.18 | 2.67 | 0.00034 | 0.0036 |
| **Inflammatory/immune** | 61 | 9 | 3.70 | 2.43 | 2.83 | 0.0096 | 0.067 |
| **Neurological/psychiatric/sleep** | 67 | 9 | 4.06 | 2.22 | 2.53 | 0.0175 | 0.092 |
| **Gastrointestinal** | 22 | 4 | 1.33 | 3.00 | 3.53 | 0.0400 | 0.17 |
| **Socioeconomic** | 9 | 1 | 0.55 | 1.83 | 1.95 | 0.43 | 1 |
| **Medical history/misc coding** | 25 | 2 | 1.52 | 1.32 | 1.35 | 0.45 | 1 |
| **Bone/musculoskeletal** | 45 | 3 | 2.73 | 1.10 | 1.11 | 0.52 | 1 |
| **Cardiovascular** | 247 | 15 | 14.97 | 1.00 | 1.00 | 0.54 | 1 |
| **Reproductive/hormonal** | 32 | 2 | 1.94 | 1.03 | 1.03 | 0.59 | 1 |
| **Kidney/urinary** | 108 | 6 | 6.55 | 0.92 | 0.91 | 0.65 | 1 |
| **Other blood cell traits** | 129 | 5 | 7.82 | 0.64 | 0.61 | 0.91 | 1 |
| **Biochemistry/nutrients** | 69 | 2 | 4.18 | 0.48 | 0.45 | 0.93 | 1 |
| **Respiratory** | 51 | 1 | 3.09 | 0.32 | 0.30 | 0.96 | 1 |
| **Anthropometric/adiposity** | 165 | 5 | 10 | 0.50 | 0.46 | 0.98 | 1 |
| **Metabolic/diabetes** | 106 | 2 | 6.42 | 0.31 | 0.29 | 0.99 | 1 |
| **Lipids** | 136 | 1 | 8.24 | 0.12 | 0.11 | 0.99 | 1 |
| **Other** | 323 | 6 | 19.58 | 0.31 | 0.25 | 0.99 | 1 |
| **Behavioural/lifestyle** | 35 | 0 | 2.12 | 0 | 0 | 1 | 1 |
| **Liver/biochemistry** | 20 | 0 | 1.21 | 0 | 0 | 1 | 1 |
| **Sensory/eye/ear** | 15 | 0 | 0.91 | 0 | 0 | 1 | 1 |

*nominally significant prior to Bonferroni correction. ^#^ Grouping as detailed below
PGS, Polygenic risk score; sig, significant

**Biological trait domains definitions:**
results <- results %>% mutate( Trait_lower = str_to_lower(Trait),Trait_Group = case_when (
str_detect(Trait_lower, "platelet|thrombocyte") ~ "Platelet",
str_detect(Trait_lower, "red blood|erythrocyte|reticulocyte|hemoglobin|haemoglobin|hematocrit|haematocrit|mean corpuscular|rbc") ~ "Other blood cell traits",
str_detect(Trait_lower, "white blood|leukocyte|neutrophil|lymphocyte|monocyte|eosinophil|basophil|myeloid") ~ "Other blood cell traits",
str_detect(Trait_lower, "coronary|cardiovascular|heart|myocardial|angina|atrial fibrillation|stroke|aneurysm|vascular|qt interval|qtc|pulse|cardiac|hypertension|blood pressure") ~ "Cardiovascular",
str_detect(Trait_lower, "cholesterol|hdl|ldl|triglyceride|lipid|apolipoprotein|nonhdl") ~ "Lipids",
str_detect(Trait_lower, "diabetes|glucose|insulin|hba1c|glycated|metabolic syndrome") ~ "Metabolic/diabetes",
str_detect(Trait_lower, "body mass|bmi|adiposity|obesity|waist|hip|height|weight|fat mass|fat percentage|lean mass|body size|visceral adipose|abdominal adipose") ~ "Anthropometric/adiposity",
str_detect(Trait_lower, "cancer|carcinoma|melanoma|tumor|tumour|neoplasm|leukemia|lymphoma|myeloma|polyp") ~ "Cancer",
str_detect(Trait_lower, "alzheimer|dementia|parkinson|multiple sclerosis|epilepsy|neuroticism|depression|bipolar|schizophrenia|adhd|autism|cognitive|mental|psychiatric|insomnia|sleep") ~ "Neurological/psychiatric/sleep",
str_detect(Trait_lower, "asthma|copd|pulmonary|lung function|fev|fvc|respiratory|bronchitis") ~ "Respiratory",
str_detect(Trait_lower, "inflammatory|immune|autoimmune|ibd|crohn|ulcerative|eczema|psoriasis|allergy|rheumatoid|lupus|coeliac|celiac") ~ "Inflammatory/immune",
str_detect(Trait_lower, "kidney|renal|egfr|glomerular|creatinine|cystatin|urea|urinary|urine") ~ "Kidney/urinary",
str_detect(Trait_lower, "liver|alanine aminotransferase|aspartate aminotransferase|alkaline phosphatase|bilirubin|ggt|gamma|cirrhosis") ~ "Liver/biochemistry",
str_detect(Trait_lower, "albumin|calcium|phosphate|protein|vitamin|urate|uric acid|iron|ferritin|biomarker|enzyme") ~ "Biochemistry/nutrients",
str_detect(Trait_lower, "bone|osteoporosis|arthrosis|arthritis|osteoarthritis|fracture|heel|bmd|mineral density|muscle|musculoskeletal") ~ "Bone/musculoskeletal",
str_detect(Trait_lower, “menopause|menarche|birth|pregnancy|endometriosis|ovarian|uterine|prostate|testosterone|reproductive|sexual") ~ "Reproductive/hormonal",
str_detect(Trait_lower, "appendicitis|appendectomy|diverticular|gallstone|cholelithiasis|intestinal|bowel|stomach|gastric|abdominal pain|digestive|gastro") ~ "Gastrointestinal",
str_detect(Trait_lower, "smoking|alcohol|diet|coffee|tea|water intake|physical activity|walking|sedentary|lifestyle") ~ "Behavioural/lifestyle",
str_detect(Trait_lower, "education|income|occupation|employment|social|household|deprivation") ~ "Socioeconomic",
str_detect(Trait_lower, "eye|vision|glasses|macular|retina|cataract|hearing") ~ "Sensory/eye/ear",
str_detect(Trait_lower, "^z[0-9]|^i[0-9]|^n[0-9]|^k[0-9]|phecode|history of|encountering health services|implants|screen displayed") ~ "Medical history/misc coding",
TRUE ~ "Other"))

**Table S3. Outcome risk of aspirin vs placebo, within platelet polygenic score quintile stratification**

|  | **Dementia** | | | | | **Major Bleeding** | | | |
| --- | --- | --- | --- | --- | --- | --- | --- | --- | --- |
| **PGS ID** | **Quintile** | **Events** | **HR** | **95%CI** | **p-value*** | **Events** | **HR** | **95%CI** | **p-value*** |
| **PGS001971** | Q1 | 73 | 1.49 | 0.93-2.40 | 0.098 | 77 | 1.09 | 0.69-1.71 | 0.72 |
|  | Q2 | 71 | 1.28 | 0.80-2.05 | 0.31 | 84 | 1.45 | 0.94-2.24 | 0.096 |
|  | Q3 | 70 | 0.74 | 0.46-1.20 | 0.22 | 90 | 0.87 | 0.57-1.32 | 0.52 |
|  | Q4 | 75 | 0.75 | 0.47-1.19 | 0.22 | 67 | 1.41 | 0.87-2.30 | 0.17 |
|  | Q5 | 56 | **0.43** | 0.24-0.77 | **0.0044** | 96 | **1.94** | 1.27-2.97 | **0.0022** |
|  | | | | | | | | | |
| **PGS002189** | Q1 | 77 | **1.62** | 1.02-2.59 | **0.04** | 72 | 1.18 | 0.74-1.89 | 0.49 |
|  | Q2 | 73 | 1.23 | 0.77-1.96 | 0.38 | 87 | 1.46 | 0.95-2.24 | 0.082 |
|  | Q3 | 62 | 0.89 | 0.54-1.46 | 0.63 | 92 | 0.73 | 0.48-1.11 | 0.14 |
|  | Q4 | 81 | 0.66 | 0.42-1.04 | 0.072 | 75 | **1.67** | 1.05-2.68 | **0.031** |
|  | Q5 | 52 | **0.30** | 0.16-0.57 | **0.00022** | 88 | **1.97** | 1.27-3.07 | **0.0027** |
|  | | | | | | | | | |
| **PGS003548** | Q1 | 76 | 1.45 | 0.91-2.32 | 0.12 | 72 | 0.95 | 0.60-1.51 | 0.83 |
|  | Q2 | 75 | 1.15 | 0.73-1.81 | 0.55 | 85 | 1.32 | 0.86-2.02 | 0.20 |
|  | Q3 | 62 | 0.96 | 0.58-1.58 | 0.86 | 92 | 1.23 | 0.81-1.85 | 0.33 |
|  | Q4 | 73 | 0.80 | 0.50-1.27 | 0.34 | 78 | 1.08 | 0.69-1.68 | 0.75 |
|  | Q5 | 59 | **0.28** | 0.15-0.52 | **5.84E-05** | 87 | **2.13** | 1.36-3.34 | **0.00092** |

*Adjusted for age, sex, number of APOE ε3 and APOE ε4 alleles, and top 10 genetic principal components

**Table S4. Outcome risk of aspirin vs placebo, within platelet polygenic score quintile stratification (full adjustment)**

|  | **Dementia** | | | | | **Major Bleeding** | | | |
| --- | --- | --- | --- | --- | --- | --- | --- | --- | --- |
| **PGS ID** | **Quintile** | **Events** | **HR** | **95%CI** | **p-value*** | **Events** | **HR** | **95%CI** | **p-value*** |
| **PGS001971** | Q1 | 73 | 1.39 | 0.86-2.25 | 0.18 | 77 | 1.06 | 0.68-1.67 | 0.80 |
|  | Q2 | 71 | 1.09 | 0.67-1.76 | 0.73 | 84 | 1.48 | 0.95-2.28 | 0.08 |
|  | Q3 | 70 | 0.68 | 0.41-1.10 | 0.16 | 90 | 0.88 | 0.58-1.33 | 0.54 |
|  | Q4 | 75 | 0.94 | 0.59-1.51 | 0.80 | 67 | 1.40 | 0.86-2.29 | 0.18 |
|  | Q5 | 56 | **0.44** | 0.24-0.79 | **0.0059** | 96 | **1.92** | 1.25-2.93 | **0.003** |
|  | | | | | | | | | |
| **PGS002189** | Q1 | 77 | 1.52 | 0.95-2.44 | 0.08 | 72 | 1.17 | 0.73-1.87 | 0.52 |
|  | Q2 | 73 | 1.06 | 0.66-1.69 | 0.81 | 87 | 1.48 | 0.97-2.28 | 0.07 |
|  | Q3 | 62 | 0.86 | 0.51-1.43 | 0.56 | 92 | 0.72 | 0.48-1.10 | 0.13 |
|  | Q4 | 81 | 0.75 | 0.47-1.19 | 0.22 | 75 | **1.70** | 1.06-2.73 | **0.03** |
|  | Q5 | 52 | **0.32** | 0.17-0.61 | **0.00053** | 88 | **1.94** | 1.24-3.03 | **0.004** |

*Adjusted for age, sex, baseline smoking status, alcohol consumption, body-mass index, hypertension, diabetes, 3MS score and education level (≤12 years vs ≥13 years), *APOE* ε2 count and *APOE* ε4 allele count, high density lipoprotein, low density lipoprotein, prior use of aspirin or anti-inflammatory drugs and top 10 genetic principal components.

**Table S5. Comparison of baseline characteristics and endpoints between aspirin vs placebo allocated participants within PGS001971 and PGS002189 Quintile 5.**

| **Baseline characteristics** | **PGS001971 (Quintile 5)**  **(n=2708)** | | | **PGS002189 (Quintile 5)**  **(n=2708)** | | |
| --- | --- | --- | --- | --- | --- | --- |
|  | **Aspirin**  **(n=1331)** | **Placebo  (n=1377)** | **p-value** | **Aspirin**  **(n=1333)** | **Placebo  (n=1375)** | **p-value** |
| **Age, mean (SD)** | 75.2 (4.5) | 75.0 (4.3) | 0.27 | 75.1 (4.4) | 74.8 (4.3) | 0.11 |
| **Sex female, n (%)** | 739 (55.5) | 738 (53.6) | 0.31 | 741 (55.6) | 751 (54.6) | 0.61 |
| **BMI (kg/m^2^), mean (SD)** | 28.3 (4.5) | 28.1 (4.7) | 0.19 | 28.3 (4.6) | 28.1 (4.6) | 0.36 |
| **Hypertension, n (%)** | 1003 (75.4) | 1034 (75.1) | 0.91 | 993 (74.5) | 1021 (74.3) | 0.92 |
| **Diabetes, n (%)** | 128 (9.6) | 150 (10.9) | 0.30 | 118 (8.9) | 144 (10.5) | 0.17 |
| **Baseline 3MS, mean (SD)** | 93.7 (4.4) | 93.8 (4.3) | 0.63 | 93.7 (4.4) | 93.8 (4.3) | 0.73 |
| **Education** ≥**13 years, n (%)** | 542 (40.7) | 600 (43.6) | 0.14 | 535 (40.1) | 586 (42.6) | 0.20 |
| **Smoking status, n (%)** |  |  |  |  |  |  |
| Current/Former | 610 (45.8) | 610 (44.3) | 0.44 | 594 (44.6) | 621 (45.2) | 0.78 |
| Never | 721 (54.2) | 767 (55.7) |  | 739 (55.4) | 754 (54.8) |  |
| **Alcohol use, n (%)** |  |  |  |  |  |  |
| Current | 1059 (79.6) | 1101 (80) | 0.37 | 1057 (79.3) | 1100 (80) | 0.68 |
| Former/Never | 272 (20.4) | 276 (20) |  | 276 (20.7) | 275 (20) |  |
| **Medication** |  |  |  |  |  |  |
| Statins, n (%) | 390 (29.3) | 391 (28.4) | 0.63 | 389 (29.2) | 405 (29.5) | 0.91 |
| NSAID, n (%) | 171 (12.9) | 191 (13.9) | 0.47 | 171 (12.8) | 192 (14.0) | 0.42 |
| Previous aspirin use, n (%) | 126 (9.5) | 123 (8.9) | 0.68 | 122 (9.2) | 125 (9.1) | 0.99 |
| **Cholesterol** |  |  |  |  |  |  |
| HDL (mmol/L), mean (SD) | 1.59 (0.5) | 1.57 (0.4) | 0.43 | 1.58 (0.5) | 1.58 (0.4) | 0.97 |
| LDL (mmol/L), mean (SD) | 3.08 (0.87) | 3.07 (0.85) | 0.75 | 3.09 (0.88) | 3.06 (0.85) | 0.37 |
| ***APOE* genotype** |  |  |  |  |  |  |
| ε1/ε3: ε2/ε4, n (%) | 39 (2.9) | 50 (33.6) | 0.29 | 41 (3.1) | 44 (3.2) | 0.88 |
| ε1/ε4, n (%) | 0 (0) | 0 (0) |  | 0 (0) | 0 (0) |  |
| ε2/ε2, n (%) | 11 (0.8) | 4 (0.3) |  | 11 (0.8) | 7 (0.5) |  |
| ε2/ε3, n (%) | 205 (15.4) | 195 (14.2) |  | 192 (14.4) | 201 (14.6) |  |
| ε3/ε3, n (%) | 793 (59.6) | 849 (61.7) |  | 804 (60.3) | 846 (61.5) |  |
| ε3/ε4, n (%) | 268 (20.1) | 262 (19.0) |  | 269 (20.2) | 261 (19) |  |
| ε4/ε4, n (%) | 15 (1.1) | 17 (1.2) |  | 16 (1.2) | 16 (1.2) |  |
|  |  |  |  |  |  |  |
| **Endpoints, n (%)** |  |  |  |  |  |  |
| Dementia | 17 (1.3) | 39 (2.8) | **0.0068** | 13 (1.0) | 39 (2.8) | **0.0007** |
| Major Bleeding | 63 (4.7) | 33 (2.4) | **0.0015** | 58 (4.4) | 30 (2.2) | **0.0021** |

BMI, body mass index; HDL, high density lipoprotein; LDL, low density lipoprotein; NSAID, non-steroidal anti-inflammatory drug use.

**Table S6. Stratification sensitivity analyses using alternative genetic risk thresholds across Bonferroni-significant platelet-related PGS.**

| **PGS** | **Stratification** | **N** | **Dementia**  **events** | **N_aspirin** | **N_placebo** | **Events**  **aspirin** | **Events**  **placebo** | **HR** | **95%CI** | **P_value** |
| --- | --- | --- | --- | --- | --- | --- | --- | --- | --- | --- |
| **PGS003548** | Top quintile (Q5) | 2708 | 59 | 1310 | 1398 | 13 | 46 | 0.28 | 0.15 – 0.52 | 5.82E-05 |
|  | Top decile (D10) | 1354 | 22 | 668 | 686 | 2 | 20 | 0.09 | 0.02 – 0.41 | 0.001826 |
|  | Top 5% | 678 | 14 | 344 | 334 | 1 | 13 | 0.07 | 0.01 – 0.58 | 0.014153 |
|  | Top tertile (T3) | 4513 | 111 | 2212 | 2301 | 36 | 75 | 0.48 | 0.32 – 0.72 | 0.000321 |
| **PGS002189** | Top quintile (Q5) | 2708 | 52 | 1333 | 1375 | 13 | 39 | 0.30 | 0.16 – 0.57 | 0.000222 |
|  | Top decile (D10) | 1354 | 23 | 672 | 682 | 2 | 21 | 0.08 | 0.02 – 0.37 | 0.000986 |
|  | Top 5% | 678 | 14 | 337 | 341 | 1 | 13 | 0.04 | 0.01 – 0.37 | 0.003926 |
|  | Top tertile (T3) | 4513 | 103 | 2209 | 2304 | 34 | 69 | 0.50 | 0.33 – 0.76 | 0.001036 |
| **PGS001971** | Top quintile (Q5) | 2708 | 56 | 1331 | 1377 | 17 | 39 | 0.43 | 0.24 – 0.77 | 0.004444 |
|  | Top decile (D10) | 1354 | 27 | 666 | 688 | 4 | 23 | 0.17 | 0.06 – 0.51 | 0.001675 |
|  | Top 5% | 678 | 13 | 335 | 343 | 1 | 12 | 0.08 | 0.01 – 0.62 | 0.015943 |
|  | Top tertile (T3) | 4513 | 102 | 2231 | 2282 | 34 | 68 | 0.49 | 0.32 – 0.74 | 0.000652 |
